# (^S^ *S*,2*S*,3*S*)-2-(2-Methyl­propan-2-sulfin­amido)-3-phenyl­butyronitrile

**DOI:** 10.1107/S1600536809041245

**Published:** 2009-10-17

**Authors:** Klaus Harms, Michael Marsch, Markus Oberthür, Peter Schüler

**Affiliations:** a Philipps-Universität Marburg, Fachbereich Chemie, Hans-Meerwein-Strasse, D-35032 Marburg, Germany

## Abstract

The absolute configuration has been determined for the title compound, C_14_H_20_N_2_OS. There are two independent mol­ecules in the asymmetric unit. Inter­molecular N—H⋯O hydrogen bonds are observed in the crystal packing, forming infinite chains with the base vectors [100] and [010]. Each chain contains only one of the two independent mol­ecules.

## Related literature

For uses of *tert*-butane­sulfinimines, see: Ferreira *et al.* (2009 ▶). For asymmetric Strecker reactions utilizing this auxiliary, see: Davis *et al.* (1994 ▶); Li *et al.* (2003 ▶). For natural sources of (2*S*,3*S*)-β-methyl­phenyl­alanine, see: Singh *et al.* (2003 ▶); Kaneda (1992 ▶, 2002 ▶). For a related structure, see: Harms *et al.* (2009 ▶).
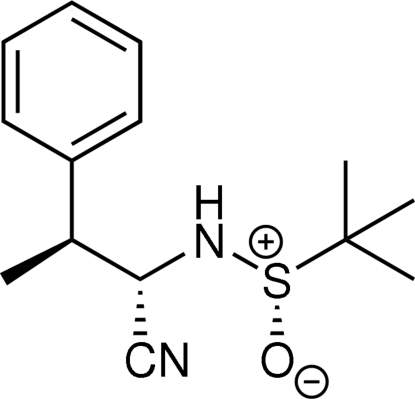


## Experimental

### 

#### Crystal data

C_14_H_20_N_2_OS
*M* *_r_* = 264.38
Orthorhombic, 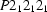

*a* = 9.0344 (4) Å
*b* = 9.0617 (5) Å
*c* = 35.767 (3) Å
*V* = 2928.1 (3) Å^3^
*Z* = 8
Mo *K*α radiation
μ = 0.21 mm^−1^
*T* = 100 K
0.36 × 0.08 × 0.06 mm

#### Data collection

Stoe IPDS II diffractometer
Absorption correction: multi-scan (Blessing, 1995 ▶) *T*_min_ = 0.936, *T*_max_ = 1.041
15474 measured reflections
5160 independent reflections
3413 reflections with *I* > 2σ(*I*)
*R*_int_ = 0.093

#### Refinement

*R*[*F* ^2^ > 2σ(*F* ^2^)] = 0.046
*wR*(*F* ^2^) = 0.074
*S* = 0.77
5160 reflections
342 parameters
2 restraints
H atoms treated by a mixture of independent and constrained refinement
Δρ_max_ = 0.19 e Å^−3^
Δρ_min_ = −0.24 e Å^−3^
Absolute structure: Flack (1983 ▶), 2183 Friedel pairs
Flack parameter: −0.04 (9)

### 

Data collection: *X-AREA* (Stoe & Cie, 2002 ▶); cell refinement: *X-AREA*; data reduction: *X-AREA*; program(s) used to solve structure: *SIR92* (Altomare *et al*., 1994 ▶); program(s) used to refine structure: *SHELXL97* (Sheldrick, 2008 ▶); molecular graphics: *DIAMOND* (Brandenburg, 1999 ▶); software used to prepare material for publication: *publCIF* (Westrip, 2008 ▶).

## Supplementary Material

Crystal structure: contains datablocks global, I. DOI: 10.1107/S1600536809041245/pv2213sup1.cif

Structure factors: contains datablocks I. DOI: 10.1107/S1600536809041245/pv2213Isup2.hkl

Additional supplementary materials:  crystallographic information; 3D view; checkCIF report

## Figures and Tables

**Table 1:** Hydrogen-bond geometry (Å, °)

*D*—H⋯*A*	*D*—H	H⋯*A*	*D*⋯*A*	*D*—H⋯*A*
N1—H1*A*⋯O1^i^	0.85 (2)	2.110 (17)	2.882 (3)	151 (3)
N21—H211⋯O21^ii^	0.85 (2)	2.23 (2)	2.995 (4)	149 (3)

Symmetry codes: (i) 
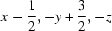
; (ii) 
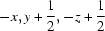
.

**Fig. 1.**
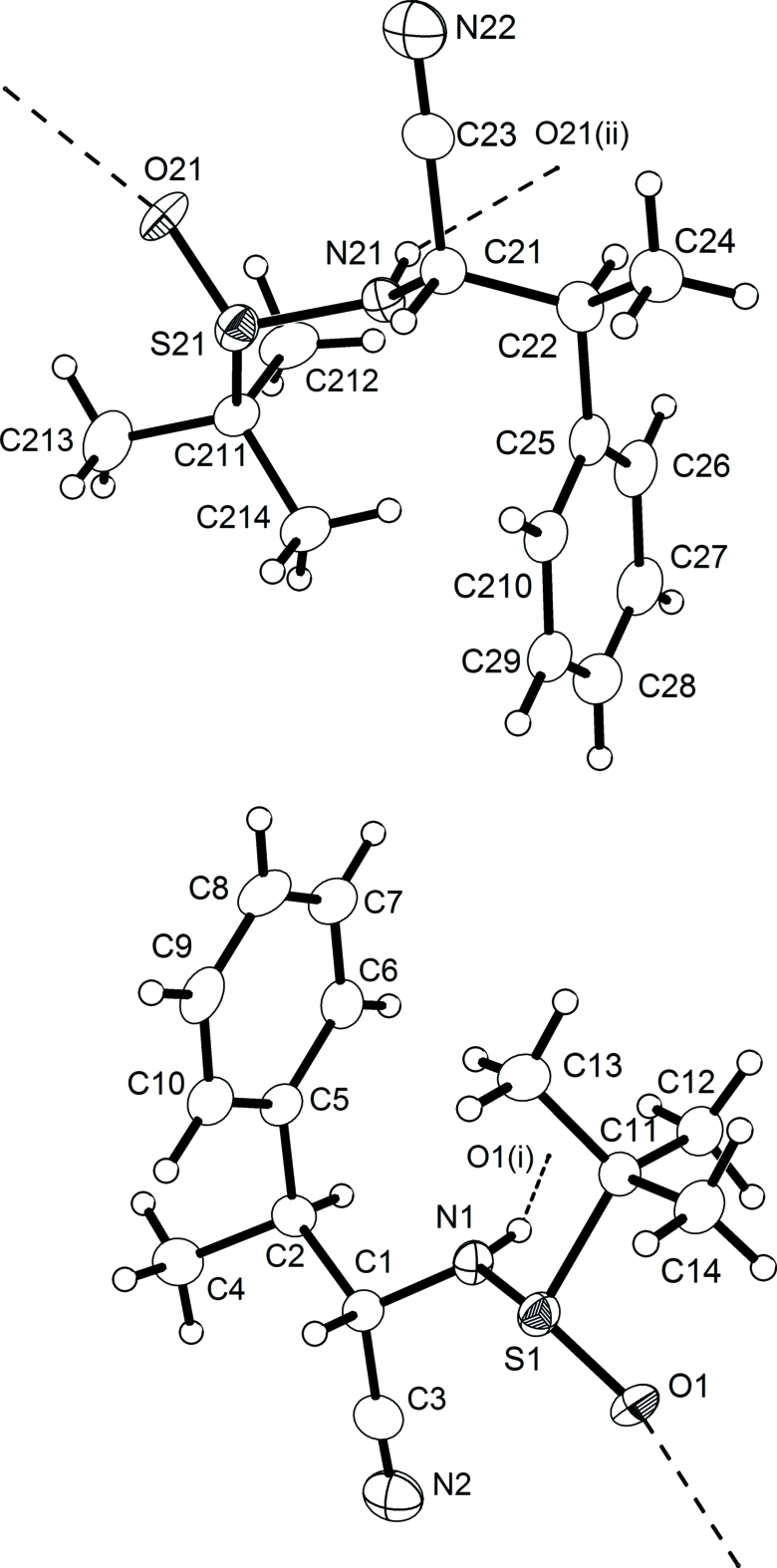
A view of the two molecules in the asymmetric unit of (I). Displacement ellipsoids are drawn at the 50% probability level. Symmetry operations, (i): x-1/2, -y+3/2, -z; (ii): -x, y+1/2, -z+1/2.

**Fig. 2.**
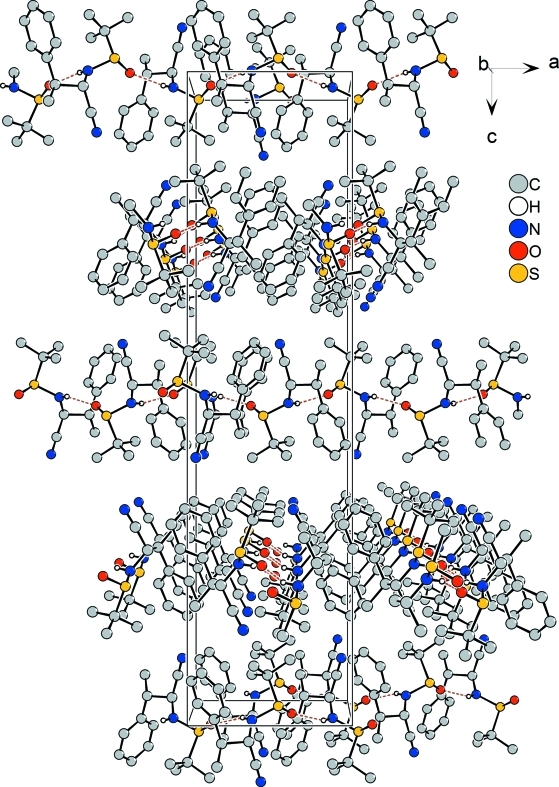
Unit cell packing of (I) viewed down the b-axis. Dotted lines indicate hydrogen bonds.

## supplementary crystallographic information

### Comment

Chiral sulfinimines have proven to be powerful and versatile precursors for the synthesis of nonproteinogenic amino acids (Ferreira *et al.*, 2008). They allow the stereoselective introduction of cyanide therefore representing an asymmetric modification of the Strecker reaction (Davis *et al.*, 1994); Li *et al.*, 2003). We have synthesized the title compound, (I), that can be hydrolyzed to give (2*S*,3*S*)-β-methylphenylalanine which is an amino acid found in the antibiotic families of the bottromycins and the mannopeptimycins (Singh *et al.*, 2003); Kaneda, 1992; and Kaneda, 2002). In this paper we report the crystal structure and absolute configuration of (I).

The molecular structure of (I) is presented in Fig. 1. There are two independent molecules in the asymmetric unit. The structure exhibits intermolecular N—H···O hydrogen bonds resulting in infinite one dimensional chains with the base vectors [1 0 0] and [0 1 0], respectively (details have have been provided in Table 1 and Fig. 2).  Each chain contains only one of the two independent molecules.

The crystal structure and absolute configuration of a closely related compound has just been reported (Harms *et al.*, 2009).

### Experimental

Trimethylsilyl cyanide (TMSCN) (706 µ*L*, 5.64 mmol) was added dropwise to a solution of (*^S^S*)-(2-phenylpropyliden)-2-methyl-2-propansulfinylimin (1.12 g, 4.70 mmol) and CsF (858 mg, 5.64 mmol) in 50 ml *n*-hexane at 243 K. The mixture was stirred at this temperature for 14 h and subsequently quenched with semisaturated aqueous NH_4_Cl solution. Extraction with EtOAc (2×50 mL) and drying of the combined organic phases (MgSO_4_) yielded the crude mixture of 3*S */ 3*R* epimers. Crystallization from petrolether/EtOAc yielded 370 mg (1.41 mmol, 35%) of a 1:1 mixture of the diastereomers. Flash column chromatography of the mother liquor yielded 80 mg (303 mmol, 6%) of the pure 3*S *isomer, which had a slightly higher *R*_f_-value (*R*_f_=0.30 in petrol ether/EtOAc 2:1) than the 3*R *isomer of which 60 mg (227 mmol, 5%) could be isolated. The remaining fractions afforded 400 mg (1.53 mmol, 32%) of a roughly 1:1 mixture of the epimers. (*^S^S*,2*S*,3*S*)-(2-Methylpropansulfinyl)-2-amino-3-phenylbutyronitrile was crystallized from petrol ether/THF.

### Refinement

The amino H atoms were isotropically refined with a restraint (0.85 Å) N—H distance. The other H atoms were positioned geometrically (C—H = 0.95–1.00 Å) and allowed to ride on their parent atoms, with 1.5 *U*_eq_(C_methyl_) or 1.2 *U*_eq_(C).

### Crystal data

**Table:**

C_14_H_20_N_2_OS	*F*(000) = 1136
*M**_r_* = 264.38	*D*_x_ = 1.199 Mg m^−^^3^
Orthorhombic, *P*2_1_2_1_2_1_	Mo *K*α radiation, λ = 0.71073 Å
Hall symbol: P 2ac 2ab	Cell parameters from 10131 reflections
*a* = 9.0344 (4) Å	θ = 2.3–25°
*b* = 9.0617 (5) Å	µ = 0.21 mm^−^^1^
*c* = 35.767 (3) Å	*T* = 100 K
*V* = 2928.1 (3) Å^3^	Prism, colourless
*Z* = 8	0.36 × 0.08 × 0.06 mm

### Data collection

**Table:**

Stoe IPDS II diffractometer	5160 independent reflections
Radiation source: sealed tube	3413 reflections with *I* > 2σ(*I*)
graphite	*R*_int_ = 0.093
area detetor, ω scans	θ_max_ = 25°, θ_min_ = 2.3°
Absorption correction: multi-scan (Blessing, 1995)	*h* = −10→10
*T*_min_ = 0.936, *T*_max_ = 1.041	*k* = −9→10
15474 measured reflections	*l* = −41→42

### Refinement

**Table:**

Refinement on *F*^2^	Hydrogen site location: CH inferred from neighbouring sites, NH located
Least-squares matrix: full	H atoms treated by a mixture of independent and constrained refinement
*R*[*F*^2^ > 2σ(*F*^2^)] = 0.046	*w* = 1/[σ^2^(*F*_o_^2^) + (0.0105*P*)^2^] where *P* = (*F*_o_^2^ + 2*F*_c_^2^)/3
*wR*(*F*^2^) = 0.074	(Δ/σ)_max_ < 0.001
*S* = 0.77	Δρ_max_ = 0.19 e Å^−^^3^
5160 reflections	Δρ_min_ = −0.24 e Å^−^^3^
342 parameters	Extinction correction: *SHELXL97* (Sheldrick, 2008)
2 restraints	Extinction coefficient: 0.0011 (3)
Primary atom site location: structure-invariant direct methods	Absolute structure: Flack (1983), 2183 Friedel pairs
Secondary atom site location: difference Fourier map	Flack parameter: −0.04 (9)

### Special details

**Table:**

Experimental. *ν*_max_/cm^-1^ 3232 (br), 2963 (w), 2930 (w), 2872(w), 1492 (w), 1454 (*m*), 1422 (*m*), 1364 (w), 1113 (w), 1085 (*m*), 1054 (*s*), 1016(*m*); δ_H _(300 MHz; DMSO) 0.75 (s, 9H, *t*Bu), 1.37 (d, 3H, ^3^*J*_Me,CH _= 7.1 Hz, CH_3_), 3.10 (dq, 1H, ^3^*J*_CH,CHN _= 10.3, *J*_CH,Me _= 7.1 Hz, CH), 4.51 (pt, 1H, ^3^*J*_CHN,CH _= 10.3 Hz, CHN), 6.24 (d, 1H, ^3^*J*_NH,CHN _= 10.5 Hz, NH), 7.14 – 7.32 (m, 5H, CH_arom_); δ_C _(75 MHz; DMSO-d_6_)18.4 (CH_3_), 21.9 (C(CH_3_)_3_), 43.2 (CH), 52.8 (CHN), 55.9 (C(CH_3_)_3_), 120.2 (CN), 126.7 (*p*-CH_arom_), 127.7 (CH_arom_), 128.2 (CH_arom_), 141.9 (*i*-C_arom_); [α]_D_^23^ -1.0 (c 1.00 in CHCl_3_).
Geometry. All s.u.'s (except the s.u. in the dihedral angle between two l.s. planes) are estimated using the full covariance matrix. The cell s.u.'s are taken into account individually in the estimation of s.u.'s in distances, angles and torsion angles; correlations between s.u.'s in cell parameters are only used when they are defined by crystal symmetry. An approximate (isotropic) treatment of cell s.u.'s is used for estimating s.u.'s involving l.s. planes.
Refinement. Refinement of *F*^2^ against ALL reflections. The weighted *R*-factor *wR* and goodness of fit *S* are based on *F*^2^, conventional *R*-factors *R* are based on *F*, with *F* set to zero for negative *F*^2^. The threshold expression of *F*^2^ > 2σ(*F*^2^) is used only for calculating *R*-factors(gt) *etc*. and is not relevant to the choice of reflections for refinement. *R*-factors based on *F*^2^ are statistically about twice as large as those based on *F*, and *R*- factors based on ALL data will be even larger.

### Fractional atomic coordinates and isotropic or equivalent isotropic displacement parameters (Å^2^)

**Table:**

	*x*	*y*	*z*	*U*_iso_*/*U*_eq_	
C21	0.2714 (4)	0.3582 (4)	0.27085 (9)	0.0236 (9)	
H21	0.36	0.2946	0.2662	0.028*	
C22	0.3222 (4)	0.5196 (4)	0.27258 (10)	0.0270 (9)	
H22	0.232	0.5812	0.2769	0.032*	
C23	0.2020 (4)	0.3113 (5)	0.30640 (10)	0.0288 (9)	
C24	0.4285 (4)	0.5485 (5)	0.30544 (10)	0.0336 (10)	
H24A	0.5189	0.4903	0.302	0.05*	
H24B	0.3805	0.5198	0.3289	0.05*	
H24C	0.4537	0.6536	0.3063	0.05*	
C25	0.3851 (4)	0.5647 (4)	0.23521 (10)	0.0251 (9)	
C26	0.3155 (4)	0.6706 (4)	0.21346 (10)	0.0310 (9)	
H26	0.229	0.7179	0.2227	0.037*	
C27	0.3702 (4)	0.7092 (4)	0.17816 (10)	0.0332 (10)	
H27	0.3217	0.783	0.1638	0.04*	
C28	0.4935 (4)	0.6406 (4)	0.16434 (11)	0.0354 (11)	
H28	0.5303	0.6659	0.1403	0.042*	
C29	0.5647 (4)	0.5341 (4)	0.18560 (10)	0.0308 (9)	
H29	0.6501	0.4861	0.176	0.037*	
C210	0.5117 (4)	0.4971 (4)	0.22081 (11)	0.0265 (9)	
H210	0.5622	0.425	0.2353	0.032*	
C211	0.0856 (4)	0.2374 (4)	0.17447 (11)	0.0281 (9)	
C212	−0.0540 (4)	0.3289 (5)	0.17864 (10)	0.0370 (10)	
H21A	−0.1007	0.3413	0.1541	0.056*	
H21B	−0.0287	0.4259	0.1889	0.056*	
H21C	−0.1227	0.2785	0.1956	0.056*	
C213	0.0538 (5)	0.0964 (5)	0.15275 (11)	0.0448 (11)	
H21D	−0.0214	0.0386	0.166	0.067*	
H21E	0.1449	0.0383	0.1505	0.067*	
H21F	0.0175	0.1216	0.1277	0.067*	
C214	0.2103 (4)	0.3255 (5)	0.15608 (10)	0.0322 (9)	
H21G	0.3006	0.2657	0.1556	0.048*	
H21H	0.2283	0.4158	0.1704	0.048*	
H21I	0.1817	0.3514	0.1305	0.048*	
N21	0.1689 (3)	0.3395 (3)	0.23975 (7)	0.0228 (7)	
N22	0.1415 (4)	0.2754 (4)	0.33308 (9)	0.0399 (9)	
O21	0.0331 (3)	0.0854 (3)	0.23728 (7)	0.0316 (6)	
S21	0.15503 (10)	0.17532 (11)	0.22032 (3)	0.0260 (2)	
C1	0.8730 (4)	0.4752 (4)	−0.02331 (10)	0.0228 (8)	
H1	0.9404	0.3911	−0.0171	0.027*	
C2	0.7114 (3)	0.4153 (4)	−0.02369 (10)	0.0252 (8)	
H2	0.6449	0.4999	−0.03	0.03*	
C3	0.9145 (4)	0.5311 (4)	−0.06101 (11)	0.0268 (9)	
C4	0.6885 (4)	0.2967 (4)	−0.05361 (10)	0.0325 (10)	
H4A	0.7481	0.2097	−0.0475	0.049*	
H4B	0.7189	0.3354	−0.078	0.049*	
H4C	0.5836	0.2691	−0.0545	0.049*	
C5	0.6698 (4)	0.3632 (4)	0.01507 (9)	0.0242 (9)	
C6	0.5653 (4)	0.4409 (4)	0.03603 (10)	0.0283 (9)	
H6	0.5181	0.5254	0.0258	0.034*	
C7	0.5311 (4)	0.3941 (5)	0.07186 (10)	0.0320 (9)	
H7	0.4586	0.4467	0.0857	0.038*	
C8	0.5988 (4)	0.2738 (4)	0.08799 (11)	0.0333 (10)	
H8	0.5752	0.2442	0.1128	0.04*	
C9	0.7019 (4)	0.1975 (4)	0.06715 (11)	0.0317 (9)	
H9	0.7496	0.1139	0.0777	0.038*	
C10	0.7372 (4)	0.2407 (4)	0.03095 (11)	0.0297 (9)	
H10	0.8079	0.1861	0.017	0.036*	
C11	0.9830 (4)	0.6723 (4)	0.07127 (9)	0.0234 (8)	
C12	0.8840 (4)	0.8074 (4)	0.06764 (10)	0.0320 (9)	
H12A	0.7922	0.78	0.0548	0.048*	
H12B	0.9355	0.8836	0.0532	0.048*	
H12C	0.8606	0.8456	0.0926	0.048*	
C13	0.9020 (4)	0.5441 (4)	0.08954 (11)	0.0317 (10)	
H13A	0.8719	0.5721	0.1149	0.047*	
H13B	0.9677	0.4582	0.0907	0.047*	
H13C	0.814	0.5194	0.0748	0.047*	
C14	1.1218 (4)	0.7103 (4)	0.09391 (10)	0.0326 (10)	
H14A	1.0935	0.7347	0.1196	0.049*	
H14B	1.1717	0.7951	0.0825	0.049*	
H14C	1.189	0.6254	0.0941	0.049*	
N1	0.8892 (3)	0.5844 (3)	0.00524 (8)	0.0215 (7)	
N2	0.9438 (4)	0.5784 (4)	−0.08972 (9)	0.0397 (8)	
O1	1.1317 (2)	0.7367 (3)	0.00919 (7)	0.0304 (6)	
S1	1.05045 (10)	0.60907 (10)	0.02574 (3)	0.0241 (2)	
H211	0.087 (2)	0.384 (4)	0.2437 (9)	0.031 (11)*	
H1A	0.831 (3)	0.658 (2)	0.0059 (9)	0.027 (10)*	

### Atomic displacement parameters (Å^2^)

**Table:**

	*U*^11^	*U*^22^	*U*^33^	*U*^12^	*U*^13^	*U*^23^
C21	0.0206 (18)	0.025 (2)	0.025 (2)	0.0023 (15)	−0.0032 (14)	0.0016 (16)
C22	0.023 (2)	0.027 (2)	0.031 (2)	0.0031 (16)	−0.0027 (16)	−0.0024 (17)
C23	0.029 (2)	0.030 (2)	0.027 (2)	0.0060 (18)	0.0026 (17)	0.001 (2)
C24	0.030 (2)	0.039 (3)	0.032 (2)	−0.0029 (19)	−0.0009 (18)	−0.0053 (18)
C25	0.027 (2)	0.021 (2)	0.0267 (19)	−0.0030 (16)	−0.0063 (16)	0.0001 (16)
C26	0.024 (2)	0.026 (2)	0.043 (2)	−0.0047 (18)	−0.0068 (16)	−0.002 (2)
C27	0.041 (2)	0.029 (2)	0.030 (2)	−0.0087 (19)	−0.0113 (19)	0.0050 (18)
C28	0.036 (2)	0.040 (3)	0.031 (2)	−0.0128 (19)	−0.0006 (17)	−0.0053 (19)
C29	0.026 (2)	0.026 (2)	0.041 (2)	−0.0049 (18)	0.0029 (19)	−0.0031 (18)
C210	0.019 (2)	0.022 (2)	0.038 (2)	−0.0009 (15)	−0.0020 (17)	0.0003 (19)
C211	0.025 (2)	0.029 (2)	0.031 (2)	−0.0043 (17)	0.0041 (16)	0.0008 (17)
C212	0.031 (2)	0.041 (2)	0.039 (2)	0.003 (2)	0.0036 (18)	0.015 (2)
C213	0.048 (2)	0.038 (3)	0.049 (3)	−0.016 (2)	−0.004 (2)	−0.002 (2)
C214	0.029 (2)	0.033 (2)	0.035 (2)	−0.0036 (19)	0.0043 (16)	0.004 (2)
N21	0.0181 (17)	0.0239 (17)	0.0263 (15)	0.0025 (14)	−0.0009 (13)	−0.0021 (14)
N22	0.039 (2)	0.038 (2)	0.043 (2)	0.0162 (17)	0.0033 (18)	0.0054 (17)
O21	0.0292 (14)	0.0269 (15)	0.0386 (15)	−0.0126 (12)	0.0023 (11)	0.0106 (13)
S21	0.0233 (5)	0.0241 (5)	0.0306 (5)	−0.0019 (4)	0.0000 (4)	0.0011 (4)
C1	0.0180 (18)	0.0209 (19)	0.030 (2)	0.0025 (14)	0.0008 (16)	0.0009 (17)
C2	0.0179 (18)	0.030 (2)	0.0282 (19)	−0.0001 (15)	−0.0006 (15)	−0.0043 (19)
C3	0.016 (2)	0.033 (2)	0.032 (2)	0.0072 (16)	−0.0033 (16)	−0.0015 (18)
C4	0.023 (2)	0.040 (3)	0.035 (2)	−0.0018 (17)	−0.0022 (16)	−0.005 (2)
C5	0.0168 (18)	0.026 (2)	0.030 (2)	−0.0042 (16)	−0.0004 (15)	−0.0012 (16)
C6	0.0199 (19)	0.027 (2)	0.038 (2)	−0.0019 (17)	−0.0033 (17)	−0.0041 (17)
C7	0.027 (2)	0.037 (2)	0.032 (2)	−0.010 (2)	0.0006 (17)	−0.002 (2)
C8	0.028 (2)	0.040 (3)	0.032 (2)	−0.0152 (18)	0.0005 (17)	0.004 (2)
C9	0.031 (2)	0.024 (2)	0.041 (2)	−0.0078 (17)	−0.0058 (17)	0.005 (2)
C10	0.0200 (19)	0.028 (2)	0.041 (2)	−0.0025 (16)	0.0004 (18)	−0.0013 (19)
C11	0.026 (2)	0.0224 (19)	0.0222 (19)	−0.0018 (16)	0.0003 (14)	−0.0002 (17)
C12	0.036 (2)	0.029 (2)	0.031 (2)	−0.0051 (18)	0.0019 (16)	−0.0009 (19)
C13	0.029 (2)	0.035 (2)	0.031 (2)	−0.0019 (17)	0.0029 (17)	0.0009 (19)
C14	0.026 (2)	0.035 (3)	0.037 (2)	−0.0069 (17)	−0.0029 (17)	−0.0021 (19)
N1	0.0145 (16)	0.0214 (18)	0.0287 (16)	0.0009 (13)	−0.0041 (12)	−0.0014 (14)
N2	0.0345 (19)	0.044 (2)	0.041 (2)	0.0121 (17)	0.0020 (17)	0.0041 (18)
O1	0.0252 (13)	0.0318 (15)	0.0342 (15)	−0.0099 (12)	0.0069 (12)	0.0021 (12)
S1	0.0194 (4)	0.0229 (5)	0.0300 (5)	−0.0018 (4)	−0.0001 (4)	−0.0006 (5)

### Geometric parameters (Å, °)

**Table:**

C21—N21	1.457 (4)	C1—N1	1.429 (4)
C21—C23	1.480 (5)	C1—C3	1.488 (5)
C21—C22	1.535 (5)	C1—C2	1.557 (4)
C21—H21	1	C1—H1	1
C22—C25	1.509 (5)	C2—C5	1.512 (5)
C22—C24	1.540 (5)	C2—C4	1.530 (5)
C22—H22	1	C2—H2	1
C23—N22	1.146 (4)	C3—N2	1.144 (4)
C24—H24A	0.98	C4—H4A	0.98
C24—H24B	0.98	C4—H4B	0.98
C24—H24C	0.98	C4—H4C	0.98
C25—C26	1.386 (5)	C5—C10	1.387 (5)
C25—C210	1.396 (5)	C5—C6	1.396 (5)
C26—C27	1.400 (5)	C6—C7	1.385 (5)
C26—H26	0.95	C6—H6	0.95
C27—C28	1.369 (5)	C7—C8	1.377 (5)
C27—H27	0.95	C7—H7	0.95
C28—C29	1.387 (5)	C8—C9	1.379 (5)
C28—H28	0.95	C8—H8	0.95
C29—C210	1.388 (5)	C9—C10	1.390 (5)
C29—H29	0.95	C9—H9	0.95
C210—H210	0.95	C10—H10	0.95
C211—C212	1.516 (5)	C11—C13	1.520 (5)
C211—C213	1.523 (5)	C11—C12	1.522 (5)
C211—C214	1.529 (5)	C11—C14	1.532 (4)
C211—S21	1.843 (4)	C11—S1	1.831 (3)
C212—H21A	0.98	C12—H12A	0.98
C212—H21B	0.98	C12—H12B	0.98
C212—H21C	0.98	C12—H12C	0.98
C213—H21D	0.98	C13—H13A	0.98
C213—H21E	0.98	C13—H13B	0.98
C213—H21F	0.98	C13—H13C	0.98
C214—H21G	0.98	C14—H14A	0.98
C214—H21H	0.98	C14—H14B	0.98
C214—H21I	0.98	C14—H14C	0.98
N21—S21	1.647 (3)	N1—S1	1.646 (3)
N21—H211	0.85 (2)	N1—H1A	0.85 (2)
O21—S21	1.498 (2)	O1—S1	1.493 (2)
			
N21—C21—C23	110.7 (3)	N1—C1—C3	112.7 (3)
N21—C21—C22	109.4 (3)	N1—C1—C2	110.1 (3)
C23—C21—C22	111.5 (3)	C3—C1—C2	110.3 (3)
N21—C21—H21	108.4	N1—C1—H1	107.9
C23—C21—H21	108.4	C3—C1—H1	107.9
C22—C21—H21	108.4	C2—C1—H1	107.9
C25—C22—C21	109.6 (3)	C5—C2—C4	112.8 (3)
C25—C22—C24	113.3 (3)	C5—C2—C1	109.5 (3)
C21—C22—C24	112.3 (3)	C4—C2—C1	112.2 (3)
C25—C22—H22	107.1	C5—C2—H2	107.3
C21—C22—H22	107.1	C4—C2—H2	107.3
C24—C22—H22	107.1	C1—C2—H2	107.3
N22—C23—C21	176.6 (4)	N2—C3—C1	177.7 (4)
C22—C24—H24A	109.5	C2—C4—H4A	109.5
C22—C24—H24B	109.5	C2—C4—H4B	109.5
H24A—C24—H24B	109.5	H4A—C4—H4B	109.5
C22—C24—H24C	109.5	C2—C4—H4C	109.5
H24A—C24—H24C	109.5	H4A—C4—H4C	109.5
H24B—C24—H24C	109.5	H4B—C4—H4C	109.5
C26—C25—C210	117.9 (4)	C10—C5—C6	118.7 (3)
C26—C25—C22	120.9 (3)	C10—C5—C2	121.1 (3)
C210—C25—C22	121.1 (3)	C6—C5—C2	120.2 (3)
C25—C26—C27	121.3 (4)	C7—C6—C5	119.6 (4)
C25—C26—H26	119.4	C7—C6—H6	120.2
C27—C26—H26	119.4	C5—C6—H6	120.2
C28—C27—C26	120.0 (4)	C8—C7—C6	122.1 (4)
C28—C27—H27	120	C8—C7—H7	119
C26—C27—H27	120	C6—C7—H7	119
C27—C28—C29	119.7 (4)	C7—C8—C9	118.1 (4)
C27—C28—H28	120.1	C7—C8—H8	121
C29—C28—H28	120.1	C9—C8—H8	121
C28—C29—C210	120.4 (4)	C8—C9—C10	121.1 (4)
C28—C29—H29	119.8	C8—C9—H9	119.4
C210—C29—H29	119.8	C10—C9—H9	119.4
C29—C210—C25	120.8 (4)	C5—C10—C9	120.4 (4)
C29—C210—H210	119.6	C5—C10—H10	119.8
C25—C210—H210	119.6	C9—C10—H10	119.8
C212—C211—C213	110.6 (3)	C13—C11—C12	111.6 (3)
C212—C211—C214	111.7 (3)	C13—C11—C14	109.8 (3)
C213—C211—C214	110.9 (3)	C12—C11—C14	110.2 (3)
C212—C211—S21	111.2 (2)	C13—C11—S1	107.7 (3)
C213—C211—S21	105.2 (3)	C12—C11—S1	111.8 (2)
C214—C211—S21	107.0 (2)	C14—C11—S1	105.5 (2)
C211—C212—H21A	109.5	C11—C12—H12A	109.5
C211—C212—H21B	109.5	C11—C12—H12B	109.5
H21A—C212—H21B	109.5	H12A—C12—H12B	109.5
C211—C212—H21C	109.5	C11—C12—H12C	109.5
H21A—C212—H21C	109.5	H12A—C12—H12C	109.5
H21B—C212—H21C	109.5	H12B—C12—H12C	109.5
C211—C213—H21D	109.5	C11—C13—H13A	109.5
C211—C213—H21E	109.5	C11—C13—H13B	109.5
H21D—C213—H21E	109.5	H13A—C13—H13B	109.5
C211—C213—H21F	109.5	C11—C13—H13C	109.5
H21D—C213—H21F	109.5	H13A—C13—H13C	109.5
H21E—C213—H21F	109.5	H13B—C13—H13C	109.5
C211—C214—H21G	109.5	C11—C14—H14A	109.5
C211—C214—H21H	109.5	C11—C14—H14B	109.5
H21G—C214—H21H	109.5	H14A—C14—H14B	109.5
C211—C214—H21I	109.5	C11—C14—H14C	109.5
H21G—C214—H21I	109.5	H14A—C14—H14C	109.5
H21H—C214—H21I	109.5	H14B—C14—H14C	109.5
C21—N21—S21	118.4 (2)	C1—N1—S1	120.2 (2)
C21—N21—H211	112 (2)	C1—N1—H1A	120 (2)
S21—N21—H211	115 (3)	S1—N1—H1A	115 (2)
O21—S21—N21	112.12 (15)	O1—S1—N1	111.33 (15)
O21—S21—C211	106.03 (15)	O1—S1—C11	105.92 (16)
N21—S21—C211	97.20 (16)	N1—S1—C11	98.29 (15)
			
N21—C21—C22—C25	−54.4 (4)	N1—C1—C2—C5	−53.4 (4)
C23—C21—C22—C25	−177.1 (3)	C3—C1—C2—C5	−178.3 (3)
N21—C21—C22—C24	178.7 (3)	N1—C1—C2—C4	−179.5 (3)
C23—C21—C22—C24	56.0 (4)	C3—C1—C2—C4	55.6 (4)
N21—C21—C23—N22	−17 (8)	N1—C1—C3—N2	−44 (10)
C22—C21—C23—N22	105 (7)	C2—C1—C3—N2	80 (10)
C21—C22—C25—C26	114.1 (4)	C4—C2—C5—C10	58.2 (4)
C24—C22—C25—C26	−119.7 (4)	C1—C2—C5—C10	−67.5 (4)
C21—C22—C25—C210	−63.4 (4)	C4—C2—C5—C6	−123.8 (3)
C24—C22—C25—C210	62.8 (4)	C1—C2—C5—C6	110.4 (3)
C210—C25—C26—C27	−0.1 (5)	C10—C5—C6—C7	−0.3 (5)
C22—C25—C26—C27	−177.7 (3)	C2—C5—C6—C7	−178.3 (3)
C25—C26—C27—C28	0.8 (5)	C5—C6—C7—C8	1.1 (5)
C26—C27—C28—C29	−0.6 (5)	C6—C7—C8—C9	−1.1 (5)
C27—C28—C29—C210	−0.2 (5)	C7—C8—C9—C10	0.2 (5)
C28—C29—C210—C25	0.9 (5)	C6—C5—C10—C9	−0.5 (5)
C26—C25—C210—C29	−0.8 (5)	C2—C5—C10—C9	177.5 (3)
C22—C25—C210—C29	176.8 (3)	C8—C9—C10—C5	0.5 (5)
C23—C21—N21—S21	−83.2 (3)	C3—C1—N1—S1	−85.7 (3)
C22—C21—N21—S21	153.6 (2)	C2—C1—N1—S1	150.7 (2)
C21—N21—S21—O21	93.0 (3)	C1—N1—S1—O1	98.6 (3)
C21—N21—S21—C211	−156.4 (2)	C1—N1—S1—C11	−150.6 (3)
C212—C211—S21—O21	59.6 (3)	C13—C11—S1—O1	−178.8 (2)
C213—C211—S21—O21	−60.2 (3)	C12—C11—S1—O1	58.2 (3)
C214—C211—S21—O21	−178.2 (2)	C14—C11—S1—O1	−61.6 (3)
C212—C211—S21—N21	−56.0 (3)	C13—C11—S1—N1	66.1 (3)
C213—C211—S21—N21	−175.7 (2)	C12—C11—S1—N1	−56.9 (3)
C214—C211—S21—N21	66.2 (3)	C14—C11—S1—N1	−176.7 (2)

### Hydrogen-bond geometry (Å, °)

**Table:**

*D*—H···*A*	*D*—H	H···*A*	*D*···*A*	*D*—H···*A*
N1—H1A···O1^i^	0.85 (2)	2.11 (2)	2.882 (3)	151 (3)
N21—H211···O21^ii^	0.85 (2)	2.23 (2)	2.995 (4)	149 (3)

Symmetry codes: (i) *x*−1/2, −*y*+3/2, −*z*; (ii) −*x*, *y*+1/2, −*z*+1/2.
